# Supplementary figures and images for: Comparative genomics analyses reveal sequence determinants underlying interspecies variations in injury-responsive enhancers
Source: BMC Genomics. 2023 Apr 5;24:177. doi: 10.1186/s12864-023-09283-8 (PMC10077677; doi:10.1186/s12864-023-09283-8)

**Figure S1**

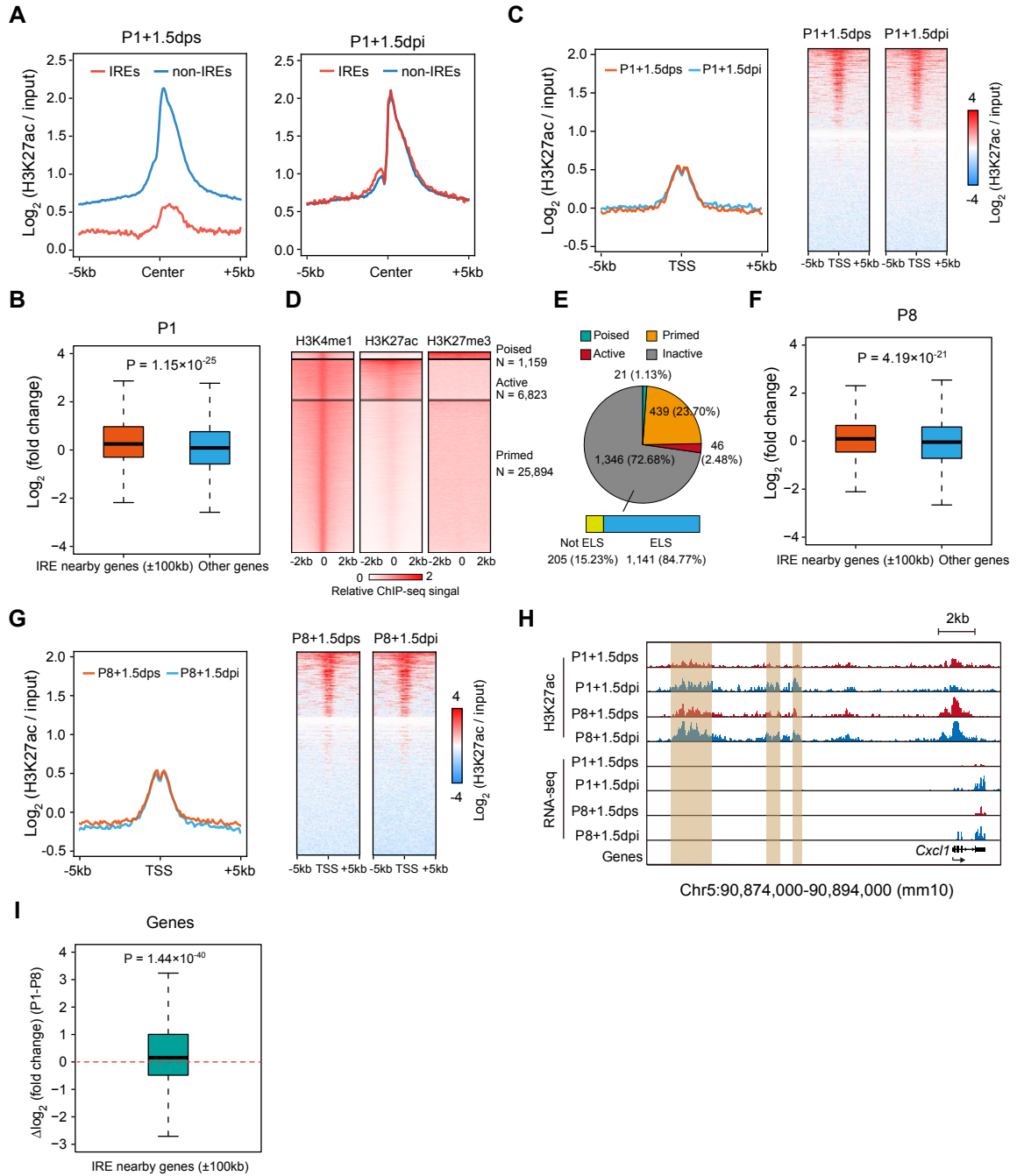

Supplement: Supplementary file 1 — Additional file 1: Fig. S1. Comparison of enhancer landscapes between mouse P1 and P8 hearts. [file 12864_2023_9283_MOESM1_ESM.pdf]

Figure S2

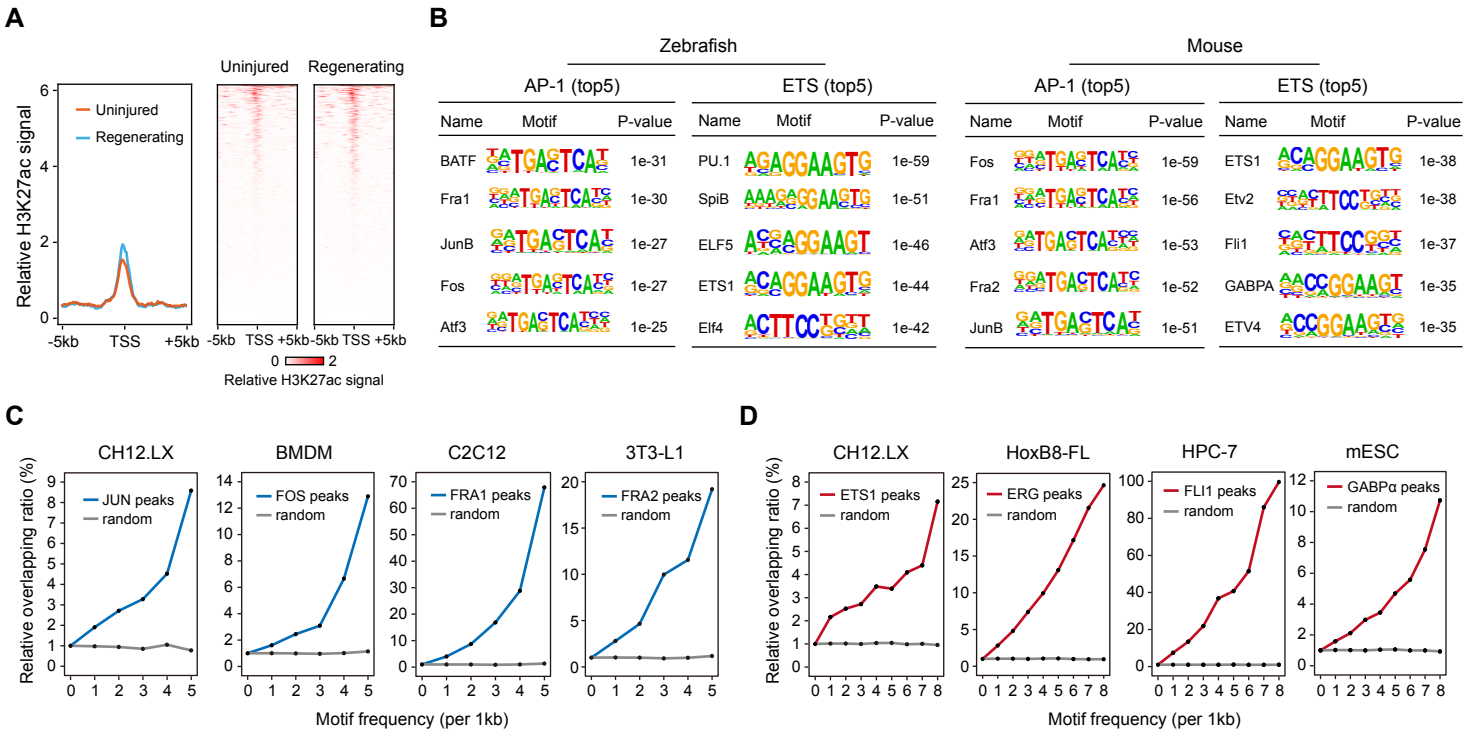

Supplement: Supplementary file 2 — Additional file 2: Fig. S2. Motif associated analyses of zebrafish and mouse cardiac IREs. [file 12864_2023_9283_MOESM2_ESM.pdf]

Figure S3

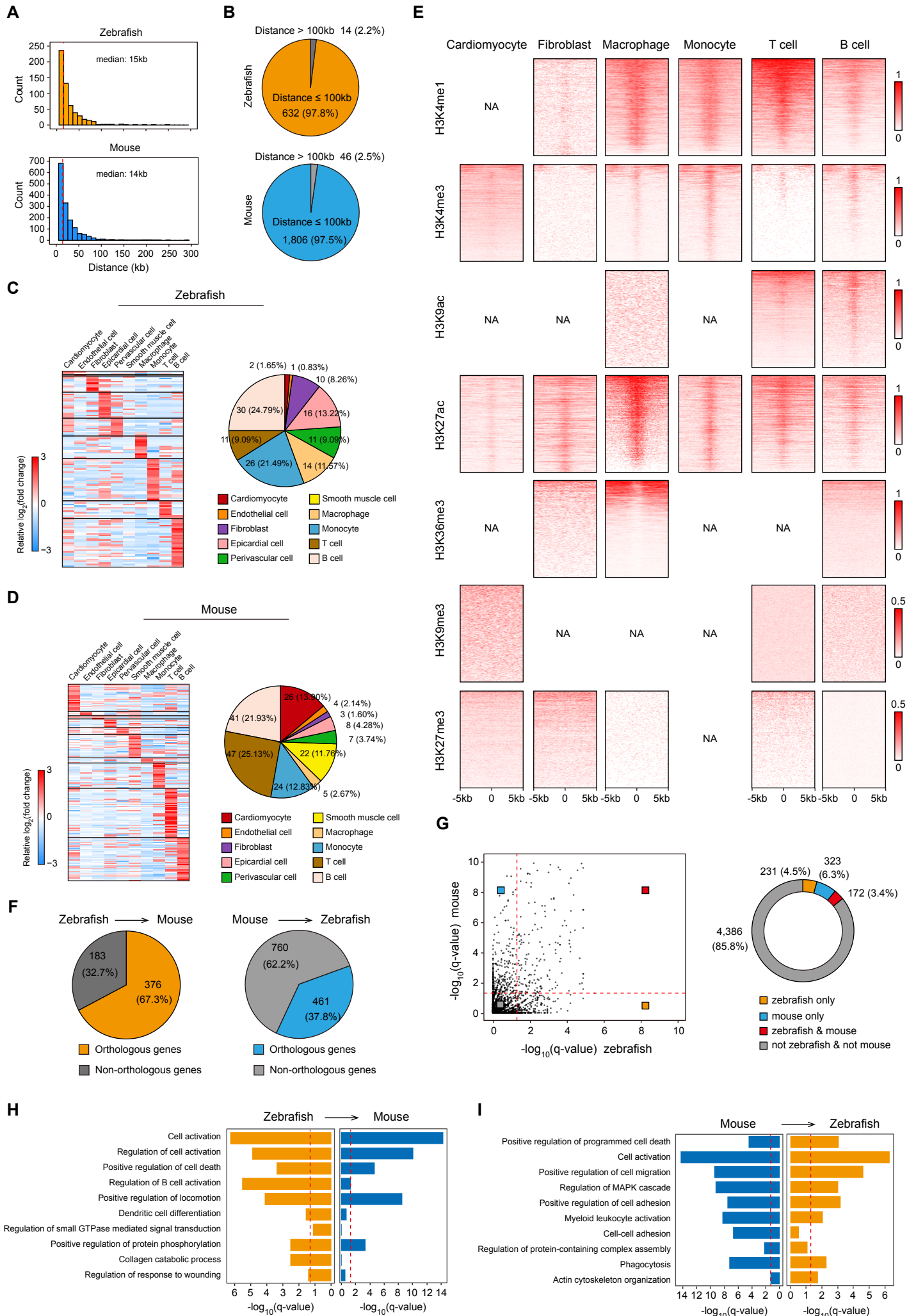

Supplement: Supplementary file 3 — Additional file 3: Fig. S3. Comparison of IRE-gene pairs between zebrafish and mice. [file 12864_2023_9283_MOESM3_ESM.pdf]

**Figure S4**

**A**

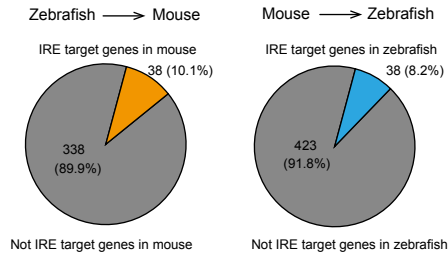

**B**

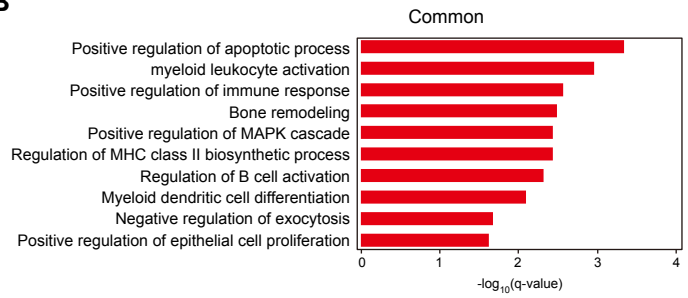

**C**

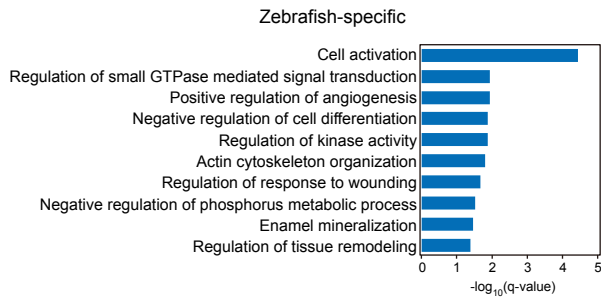

**E**

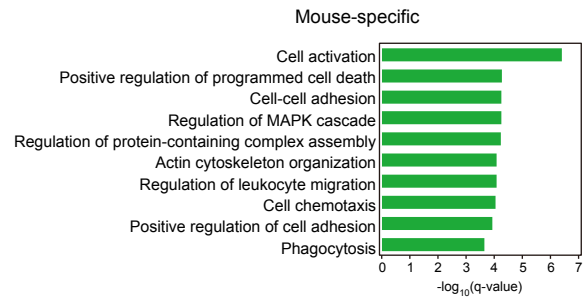

**D**

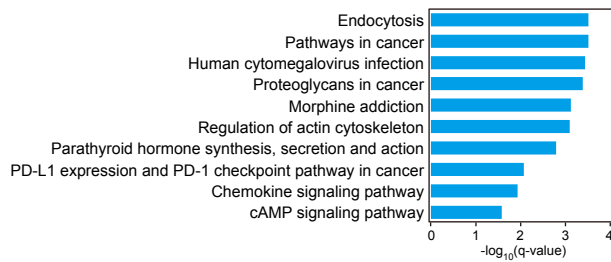

**F**

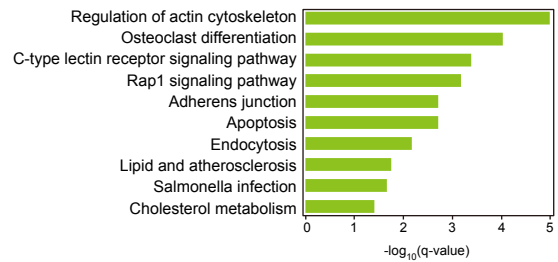

**G**

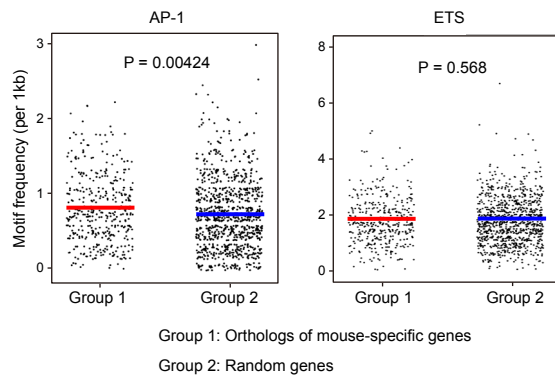

Supplement: Supplementary file 4 — Additional file 4: Fig. S4. Analyses of IRE-associated genes involved in different biological functions between zebrafish and mice. [file 12864_2023_9283_MOESM4_ESM.pdf]

Figure S5

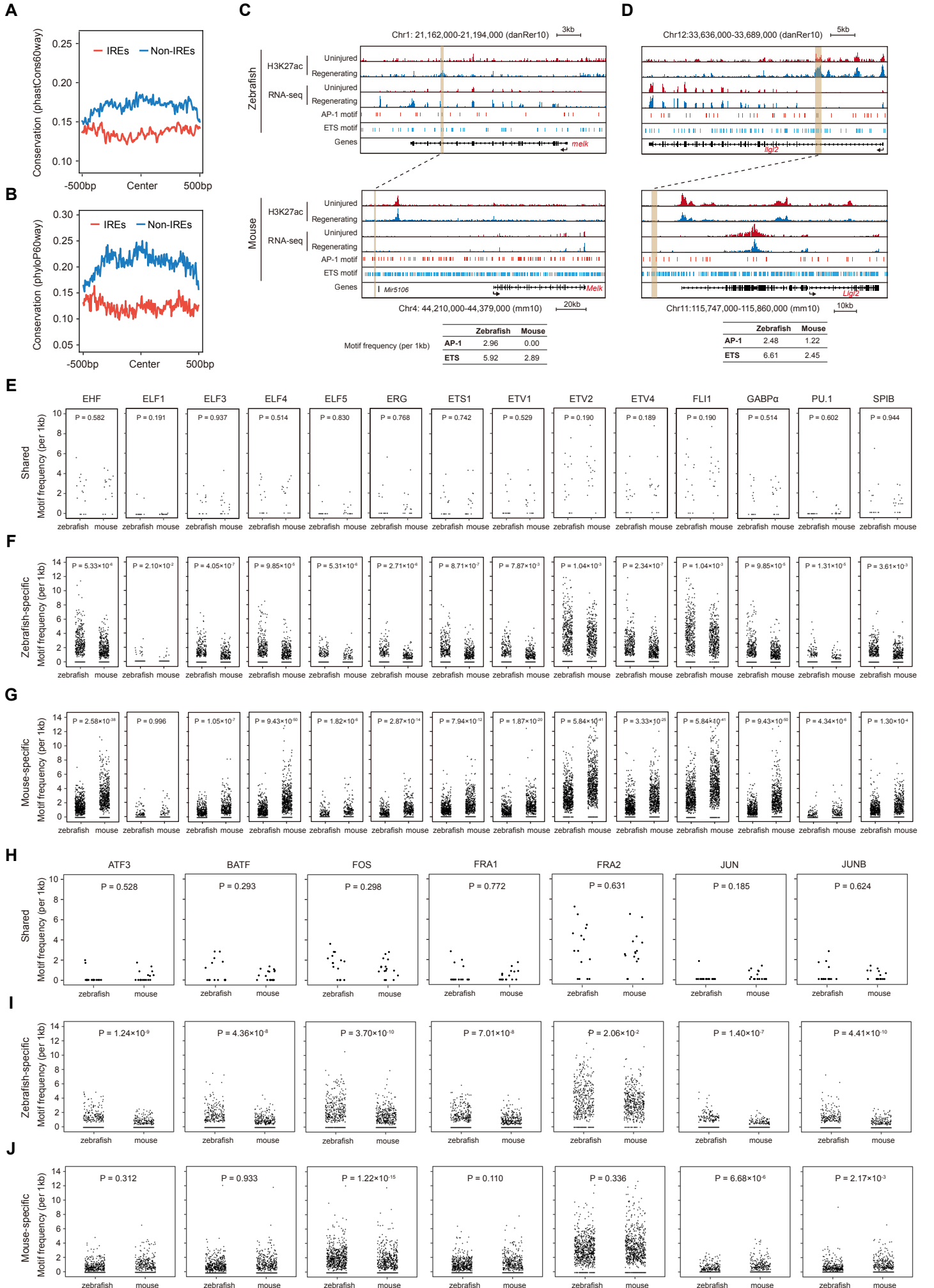

Supplement: Supplementary file 5 — Additional file 5: Fig. S5. Sequence conservation and motif frequency analyses of zebrafish and mouse cardiac IREs. [file 12864_2023_9283_MOESM5_ESM.pdf]

Figure S6

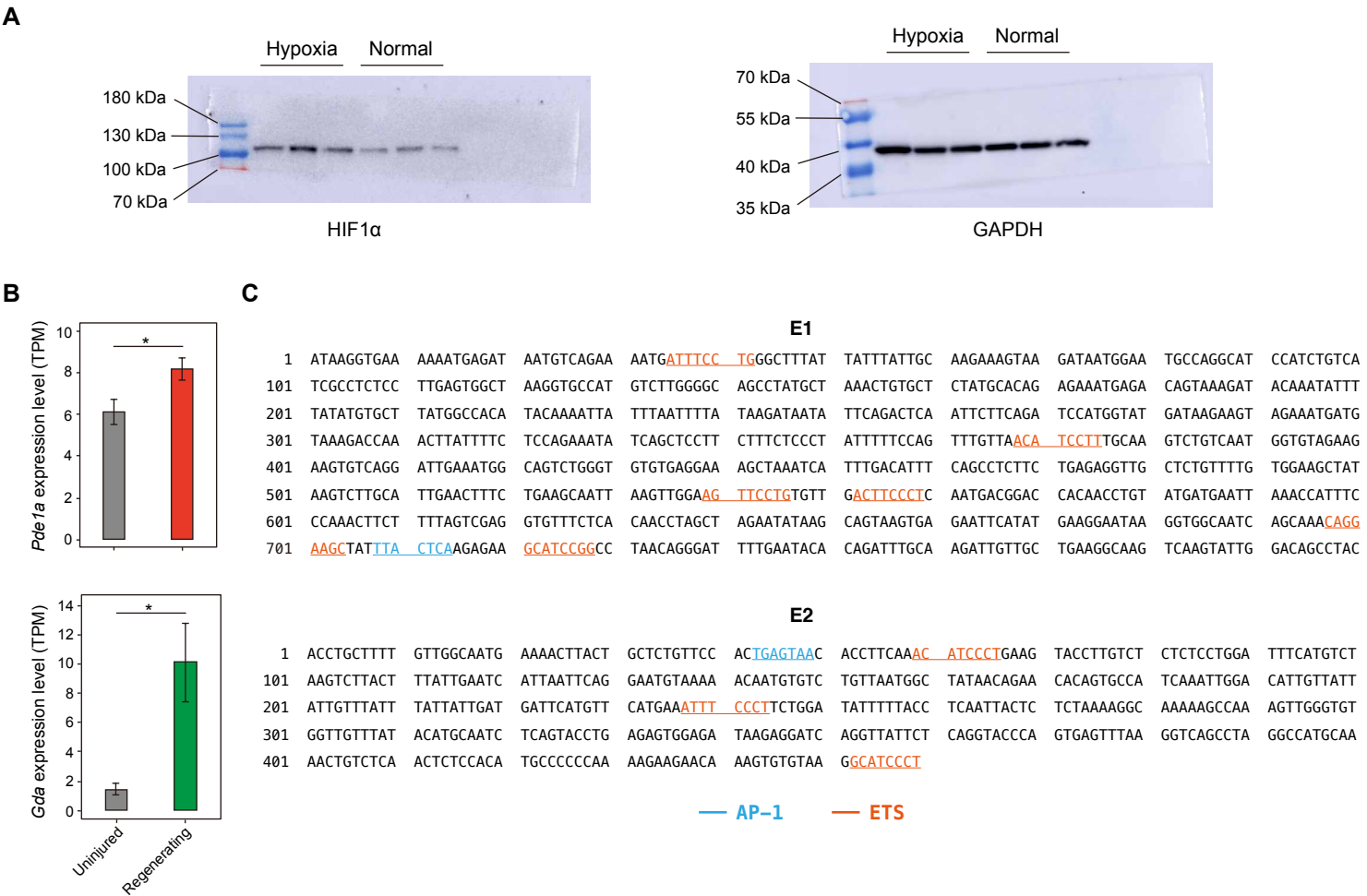

Supplement: Supplementary file 6 — Additional file 6: Fig. S6. Two mouse IREs tested in HL-1 cells with hypoxia treatment. [file 12864_2023_9283_MOESM6_ESM.pdf]
